# Phase Correction for Absorption Mode Two-Dimensional Mass Spectrometry

**DOI:** 10.3390/molecules26113388

**Published:** 2021-06-03

**Authors:** Marc-André Delsuc, Kathrin Breuker, Maria A. van Agthoven

**Affiliations:** 1 Institut de Génétique, Biologie Moléculaire et Cellulaire, INSERM U596, UMR 7104, Université de Strasbourg, 1 rue Laurent Fries, 67404 Illkirch-Graffenstaden, France; madelsuc@unistra.fr; 2 CASC4DE, Pôle API, 300 Bd. Sébastien Grant, 67400 Illkirch-Graffenstaden, France; 3 Institute for Organic Chemistry, University of Innsbruck, Innrain 80/82, 6020 Innsbruck, Austria; kathrin.breuker@uibk.ac.at

**Keywords:** mass spectrometry, two-dimensional, tandem mass spectrometry, phase correction, absorption mode, data processing

## Abstract

Two-dimensional mass spectrometry (2D MS) is a tandem mass spectrometry method that relies on manipulating ion motions to correlate precursor and fragment ion signals. 2D mass spectra are obtained by performing a Fourier transform in both the precursor ion mass-to-charge ratio (*m/z*) dimension and the fragment ion *m/z* dimension. The phase of the ion signals evolves linearly in the precursor *m/z* dimension and quadratically in the fragment *m/z* dimension. This study demonstrates that phase-corrected absorption mode 2D mass spectrometry improves signal-to-noise ratios by a factor of 2 and resolving power by a factor of 2 in each dimension compared to magnitude mode. Furthermore, phase correction leads to an easier differentiation between ion signals and artefacts, and therefore easier data interpretation.

## 1. Introduction

Two-dimensional mass spectrometry (2D MS) is a tandem mass spectrometry method that relies on manipulating ion motions to correlate precursor and fragment ion signals [1,2,3]. Various 2D MS methods have been developed for quadrupolar ion traps, linear ion traps, and Fourier transform ion cyclotron resonance mass spectrometry (FT-ICR MS) [4,5,6,7,8]. In addition to precursor-fragment correlations, two-dimensional partial correlation mass spectrometry (2D PC MS) has also been developed to explore correlations between complementary fragment ion signals [9]. In 2D FT-ICR MS, the most frequently used pulse sequence is the one proposed by Pfändler et al. to modulate ion radii in the ICR cell before fragmentation [1,2,3,10,11]. The pulse sequence and data processing have been optimized for analytical use [12,13,14,15]. 2D FT-ICR MS has been used for applications ranging from small molecules and agrochemicals, to polymers, bottom-up and top-down proteomics [16,17,18,19,20,21,22,23,24]. 2D MS has also been adapted for high-resolution analysis of precursor ions over narrower mass-to-charge ratio (*m/z*) ranges [25].

Since 2D MS relies on Fourier transformation in both dimensions, one area in which its performance can be improved is the phase correction for absorption mode mass spectrometry. Until now, 2D mass spectra have been shown in magnitude mode. In one-dimensional FT-ICR MS, phase correction for absorption mode was proposed by Xian et al. and by Qi et al. [26,27]. Automation for phase correction in one-dimensional FT-ICR MS was achieved by Kilgour et al. [28,29,30]. Processing data sets in absorption mode has improved the performance of one-dimensional FT-ICR MS by a factor of $\sqrt{2}≅1.41\;$ in signal-to-noise and a factor of 2 in resolving power, resulting in more complete and reliable analytical information for complex samples [31,32,33,34,35,36,37]. In 2D MS, we can therefore expect an improvement by a factor of $\sqrt{2}\times \sqrt{2}=2$ in signal-to-noise and in resolving power in each dimension.

A previous study has shown that the phase correction function is quadratic in the horizontal fragment ion dimension and linear in the vertical precursor ion dimension [38]. Here, we demonstrate how to perform phase correction on a narrowband 2D mass spectrum and we show how absorption mode 2D MS compares to magnitude mode.

## 2. Theory

**Scheme 1:**
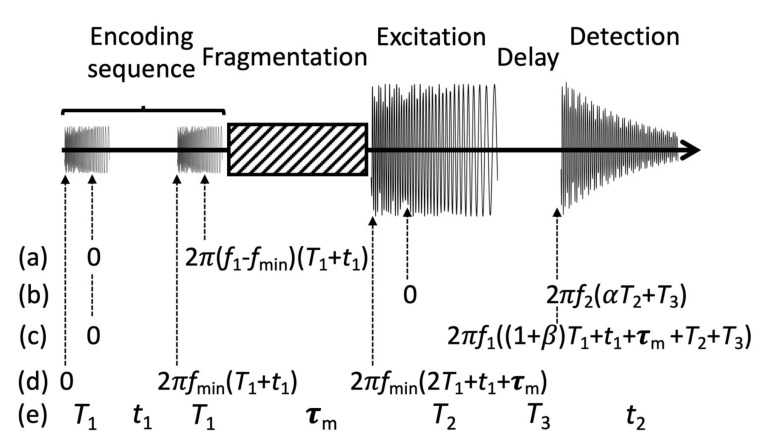
Pulse sequence for two-dimensional mass spectrometry and evolution of phases for (**a**) precursor ion radii, (**b**) fragment ion motion, (**c**) unfragmented precursor ion motion, (**d**) excitation pulses. (**e**) Duration of each segment of the pulse sequence. The factor α (resp. β) is related to the moment that the swept frequency of the chirp reaches the cyclotron frequency of the fragment (resp. unfragmented precursor).

The pulse sequence for 2D MS is shown in Scheme 1. The chirp pulses used for the excitation are needed to excite a large range of frequencies for the fragment ions and they induce a quadratic phase dependence, requiring a quadratic phase correction in the fragment ion dimension [26,27,39]. In Scheme 1, the quadratic dependence of the phase of ion signals on their resonant cyclotron frequencies is expressed through the parameters α for fragment ions and β for precursor ions. In Bruker FT-ICR mass spectrometers, the initial phase of each pulse in the pulse sequence rotates with the minimum frequency of the pulses *f*_min_. Scheme 1 shows the initial phase of each pulse, which leads to a corresponding phase offset for the signals of ions that are excited from the center of the ICR cell in that pulse. 

During the first pulse, the precursor ions are excited from the center of the ICR cell to higher radius and start accruing phase [38]. The initial phase of the first pulse is zero. At the end of the first pulse, the precursor ions have been excited to a radius *r*_0_. The precursor ions rotate during the encoding delay *t*_1_ at their own cyclotron frequency. The second pulse has a starting phase of 2π*f*_min_(*T*_1_ + *t*_1_). The phase difference between the second pulse and the precursor ion motion is 2π*t*_1_(*f*_ICR_ − *f*_min_) where *f*_ICR_ is the cyclotron frequency of the ion, *f*_min_ is the minimum frequency in the pulse, and *t*_1_ is the encoding delay. Therefore, depending on the phase difference, precursors are excited to higher radii or de-excited towards the center of the ICR cell. The frequency of the precursor ion radius modulation is *f*_ICR_ − *f*_min_. At the end of the second pulse, the radius of the precursor ions can be expressed as [2,11,40]:

(1) $$r\left(t_{1}\right)=r_{0}\sqrt{2\left(1+\cos2\pi \left(f_{\mathrm{ICR}}-f_{\min}\right)\left(t_{1}-T_{1}\right)\right)}$$

After the second pulse, a fragmentation period *τ*_m_ with a method that has a maximum fragmentation efficiency at the center of the ICR cell (e.g., electron capture dissociation, or ECD, and infrared multiphoton dissociation, or IRMPD) is applied to the precursor ions [14,22]. Ions within the fragmentation zone are fragmented. The abundance of the fragment ions is modulated with the same frequency as the radius of the precursors, i.e., *f*_ICR_–*f*_min_. The third pulse has a starting phase of 2π*f*_min_(2*T*_1_ + *t*_1_ + τ_m_) and excites all ions to high radius before detection. Fragment ions start accruing phase during the third pulse [38].

In the horizontal dimension, the phase *φ*_p,h_ accrued by precursor ions that are not fully de-excited to the center of the ICR cell at the end of the second pulse, can be expressed with a quadratic phase dependence as:

(2) $$\varphi_{p,h}\left(f_{1}\right)=c_{2}^{\prime }f_{1}^{2}+c_{1}^{\prime }f_{1}+c_{0}^{\prime }+2\pi f_{1}t_{1}\;c_{1}^{\prime }=c_{1}+T_{1}+\tau_{m}+T_{2}+T_{3}$$

in which *c*_0_′, *c*_1_′, and *c*_2_′ are the parameters for the quadratic phase incurred during excitation, *f*_1_ is the cyclotron frequency of the ion, *t*_1_ is the encoding delay, *T*_1_ is the duration of the second pulse, *τ*_m_ is the fragmentation period, *T*_2_ is the duration of the third pulse, and *T*_3_ is the delay between excitation and detection [26,27,38].

The phase *φ*_f,h_ accrued by fragment ions and unfragmented precursor ions that have been fully de-excited to the center of the ICR cell at the end of the second pulse, can be expressed with a quadratic phase dependence as:

(3) $$\varphi_{f,h}\left(f_{2}\right)=c_{2}^{\prime \prime }f_{2}^{2}+c_{1}^{\prime \prime }f_{2}+c_{0}^{\prime \prime }\;c_{0}^{\prime \prime }=c_{0}+2\pi f_{\min}\left(2T_{1}+t_{1}+\tau_{m}\right)$$

in which *c*_0_″, *c*_1_″, and *c*_2_″ are the parameters for the quadratic phase incurred during excitation, *f*_2_ is the cyclotron frequency of the ion, *t*_1_ is the encoding delay, *T*_1_ is the duration of the first and the second pulse, *τ*_m_ is the fragmentation period. As a rule, in phase-sensitive Fourier transform mass spectrometry, a shift of origin induces a zero order phase shift (*c*_0_″ in Equation (3)), a delay induces a first order phase rotation (*c*_1_″ in Equation (3)), and a frequency-dependent excitation induces a second order phase rotation (*c*_2_″ in Equation (3)).

In the vertical dimension, equation 1 shows that the phase *φ*_f,v_ for precursor ion signal can be expressed with a linear phase dependence as:

(4) $$\varphi_{p,\;v}\left(f_{1}\right)=2\pi \left(f_{1}-f_{\min}\right)\left(t_{1}-T_{1}\right)$$

in which *f*_1_ is the cyclotron frequency of the ion, *t*_1_ is the encoding delay, *f*_min_ is the lowest frequency in the pulse, and *T*_1_ is the duration of the second pulse.

Multiple studies have shown that, in the vertical dimension, for fragmentation methods that have a maximum efficiency at the center of the ICR cell, the phase *φ*_f,v_ of the fragment ion signal is shifted by π [23,24,38]. The phase of the fragment ion signal can therefore be expressed as:

(5) $$\varphi_{f,\;v}\left(f_{1}\right)=2\pi \left(f_{1}-f_{\min}\right)\left(t_{1}-T_{1}\right)+\pi$$

in which *f*_1_ is the cyclotron frequency of the precursor ion, *t*_1_ is the encoding delay, *f*_min_ is the lowest frequency in the pulse, and *T*_1_ is the duration of the second pulse.

Because signals have a *t*_1_ amplitude modulation, a data set recorded in 2D mass spectrometry experiments requires processing using hypercomplex Fourier transformation [39,40,41,42,43]. The resulting spectrum is a 4-quadrant data set:

(6) $$S\left(f_{1},\;f_{2}\right)=RR\left(f_{1},\;f_{2}\right)+iRI\left(f_{1},\;f_{2}\right)+jIR\left(f_{1},\;f_{2}\right)+kII\left(f_{1},\;f_{2}\right)$$

In which *S* is the signal in frequency domain, *f*_1_ and *f*_2_ are the frequencies, and *i*, *j*, and *k* are constants following the rules:

(7) $$i^{2}=j^{2}=-1;\;k^{2}=1$$

(8) i.j=j.i=k

(9) i.k=k.i=−j

(10) j.k=k.j=−i

With these rules, phase corrections can be applied independently to *S*(*f*_1_, *f*_2_) along the *f*_1_ and *f*_2_ axes:

(11) $$S\left(f_{1},\;f_{2}\right)e^{i\varphi_{1}}=S\left(f_{1},\;f_{2}\right)\left(\cos\varphi_{1}+i\sin\varphi_{1}\right)$$

(12) $$S\left(f_{1},\;f_{2}\right)e^{j\varphi_{2}}=S\left(f_{1},\;f_{2}\right)\left(\cos\varphi_{2}+j\sin\varphi_{2}\right)$$

The phase corrections in the horizontal dimension and in the vertical dimensions can be applied independently from each other.

## 3. Experimental Methods

### 3.1. Sample Preparation

The sample used was an equimolar mixture of unmodified and modified (mono-, di- and trimethylated) model peptides (C-terminal GK-biotinylated histone H3 sequences, AnaSpec, Fremont, CA, USA) prepared with the same protocol as described in a previous article [25]. The sequence of the histone peptides was ATKAARKSAP ATGGVKKPHR YRPGGK_biotin_.

### 3.2. Instrument Parameters

Both the standard tandem mass spectrum and the two-dimensional mass spectrum were acquired on a 7 T Apex Ultra FT-ICR mass spectrometer (Bruker Daltonik GmbH, Bremen, Germany) with an electrospray ion source operated in positive mode and direct injection at a flow rate of 70 μL/hour. In both spectra, transients were acquired for 489.34 ms (512k datapoints) with an excitation pulse before detection with a frequency range of 535,714.29–74,728.13 Hz (corresponding to *m/z* 202.22–1450). The excitation pulse was made of 739 decrements of 624 Hz with 20 μs per frequency. The amplitude of the excitation was 106 V_pp_ and the delay between excitation and detection was 3 ms.

For the standard tandem mass spectrum, ions were accumulated for 0.1 s in the first hexapole and 0.1 s in the second hexapole. In the quadrupole, ions were isolated at *m/z* 491 with an isolation window of *m/z* 30, and in the collision cell, they were activated with a 1.5 V collision energy before transfer to the Infinity ICR cell [44]. The fragmentation method was ECD, using a hollow cathode [45]. The heater was set at 1.3 A, the lens at 20 V, the bias at 1.7 V, and the irradiation lasted for 0.07 s.

The 2D mass spectrum was acquired in narrowband modulation mode [25]. The acquisition lasted 20 min. The ions were accumulated for 0.2 s in the first hexapole and 0.2 s in the second hexapole. In the quadrupole, ions were isolated at *m/z* 491 with an isolation window of *m/z* 10. In the collision cell, ions were activated with a 1.5 V collision energy. The ECD cathode was heated at 1.4 A, the lens set at 20 V, the bias at 2.0 V, and the ions were irradiated for 0.06 s. In the encoding sequence, the pulses had a frequency range of 535,714.29–74,728.13 Hz (corresponding to *m/z* 202.22–1450), with 739 decrements of 624 Hz with 1.0 μs per frequency. The amplitude of the encoding pulses was 106 V_pp_. The encoding delay incremented 1024 times by 50 μs, corresponding to a 10 kHz bandwidth, with 1 scan per increment. The signal was folded over 14 times, leading to a frequency range of 214,659.79–224,659.79 Hz (corresponding to *m/z* 482.177–504.602 for precursor ion modulation) [25]. The parameters of the excitation-detection sequence were identical to the parameters used for the standard tandem mass spectrum, with transients lasting 489.34 ms (512k datapoints).

### 3.3. Data Processing

The standard one-dimensional tandem mass spectrum and the 2D mass spectrum were processed and visualized using the Spectrometry Processing Innovative Kernel (SPIKE) software developed by the University of Strasbourg (Strasbourg, France) and CASC4DE (Illkirch-Graffenstaden, France) in the 64-bit Python 3.7 programming language on an open-source platform distributed by the Python Software Foundation (Beaverton, OR, USA) [46]. Processed data files were saved using the HDF5 file format. No denoising was applied to either dataset [12,13,15].

The transient for the one-dimensional tandem mass spectrum was apodised with a sine-bell window that was shifted, with a maximum of the bell at 15% of the transient, zero-filled twice, and Fourier transformed. The resulting spectrum was phase-corrected quadratically using a locally developed plugin to the SPIKE program (available at www.github.com/spike-project, accessed on 17 April 2021).

The program used for the data processing of the 2D mass spectrum in phase-corrected absorption mode is listed in the Appendix A. Each transient in the 2D data set was apodised with a sine-bell window that was shifted, with a maximum of the bell at 15% of the transient, zero-filled twice and Fourier transformed. The modulation induced by the frequency generator was then removed by a complex rotation of the opposite frequency in the vertical dimension along the *t*_1_ delay. The demodulation frequency was determined to be 74,659.79 Hz. The exact demodulation frequency was offset by 68 Hz from the lowest frequency entered in the Apex Control software (Bruker Daltonik GmbH, Bremen, Germany). The correct demodulation frequency was measured by processing the 2D mass spectrum with the nominal frequency entered in the software, which causes peak-splitting on the autocorrelation line, and measuring the frequency difference between the split peaks. Digital demodulation with the corrected frequency eliminated the peak-splitting [17]. The parameters determined for the one-dimensional tandem mass spectrum for quadratic phase correction were then applied to each row in the spectrum. The imaginary part of each row of the data set was then removed to reduce the computer memory burden. Then, each column of the data set was apodised with a slightly shifted sine-bell window (with a maximum of the bell at 15% of the transient), zero-filled twice and Fourier transformed. The theoretical linear phase correction determined above was applied on each column without modification. The imaginary part of the signal in each column was dropped and the dataset stored on disk.

The same 2D MS data set was processed in magnitude mode with identical apodisation and digital demodulation. However, due to the limitations of computer memory, the data-set was only zero-filled once in each dimension.

## 4. Results and Discussion

**Figure 1:**
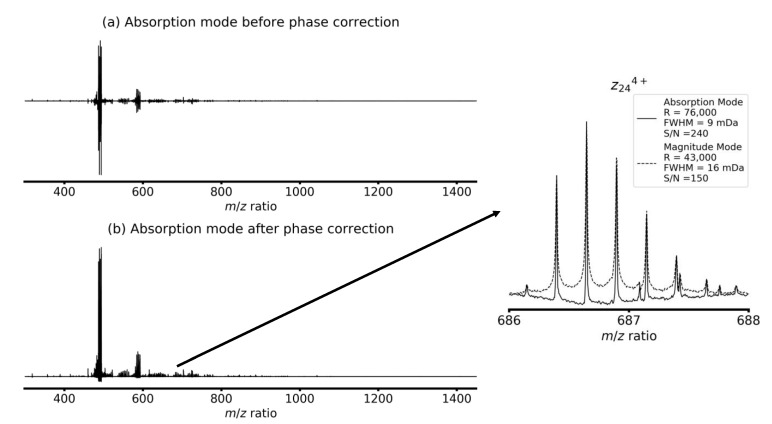
Tandem mass spectrum of the [M + 6H]^6+^ histone peptides with ECD fragmentation in absorption mode (**a**) before phase correction and (**b**) after phase correction. Insert: zoom-in on the *z*_24_^4+^ fragment ion isotopic distribution of the monomethylated histone peptide (K7 1m), comparison between magnitude mode and phase-corrected absorption mode (FWHM: full-width at half-maximum).

Figure 1 shows the result of quadratic phase correction with the SPIKE plugin for the one-dimensional ECD tandem mass spectrum of the four histone peptides. Although phase correction for FT-ICR mass spectra has been fully automated, this program requires user optimization [28,29,32,33,47]. The phase correction method is based on initial phase correction of small regions of the spectrum with linear phase functions, in a similar way to the methods proposed by Xian et al. [26] and Qi et al. [27]. The coefficients of the overall quadratic phase correction function are estimated by using the fact that these linear phase functions are tangents of the overall quadratic phase function. The user can then optimize the coefficients until the whole spectrum is properly phase-corrected.

Figure 1a shows the absorption mode mass spectrum before phase correction and Figure 1b shows the absorption mode mass spectrum after phase correction. Figure 1b shows that the signal for all the ions in the mass spectrum has been properly corrected. An insert shows a zoom-in on the isotopic distribution of the *z*_24_^4+^ fragment of the monomethylated histone peptide and compares the phase-corrected absorption mode mass spectrum to the magnitude mode mass spectrum. Both the resolving power and the signal-to-noise ratio are almost double for the phase corrected absorption mode spectrum than for the magnitude mode spectrum, as predicted by theory [26,27].

The one-dimensional tandem mass spectrum was acquired with excitation pulse parameters and an excite-detect delay that was identical to the excitation pulse and the excite-detect delay of the 2D MS pulse sequence. Qi et al. have showed that the phase correction function is very stable with experimental conditions [48]. The phase correction function determined for the one-dimensional tandem mass spectrum can therefore also be used for the phase correction of the fragment ion signals in the 2D mass spectrum, which are, analytically, the most informative signals [38].

The only difference between the excitation pulse for the tandem mass spectrum and the 2D mass spectrum is the initial phase of the pulse, which is determined by the continuous phase pulse generator. As is shown in Scheme 1, the phase shift of the excitation pulse in the 2D MS experiment is 2π*f*_min_(2*T*_1_ + *t*_1_ + *τ*_m_), in which *f*_min_ is the minimum frequency in the pulse, *T*_1_ is the duration of the first pulse, *τ*_m_ the irradiation period, and *t*_1_ is the incremental delay. The term 2π*f*_min_(2*T*_1_ + *τ*_m_) is constant throughout the experiment (and can be corrected with a constant phase offset in the phase correction function), but 2π*f*_min_*t*_1_ is different for each transient.

Digital demodulation eliminates the phase contribution of 2π*f*_min_*t*_1_ in each transient. In magnitude mode, digital demodulation is used to reduce the number of harmonics visible in the 2D mass spectrum [11,17]. Here, by applying digital demodulation before the quadratic phase correction in the horizontal dimension, we can eliminate the effect of the continuous phase pulse generator on the phase of fragment ion signals.

Table 1 gives an overview of the theoretically and empirically calculated coefficients for both phase correction functions used to calculate the absorption mode 2D mass spectrum (see Appendix A for the python code). The differences between the theoretical and calculated values shows that an operator is still needed to obtain the optimal phase correction function.

**Figure 2:**
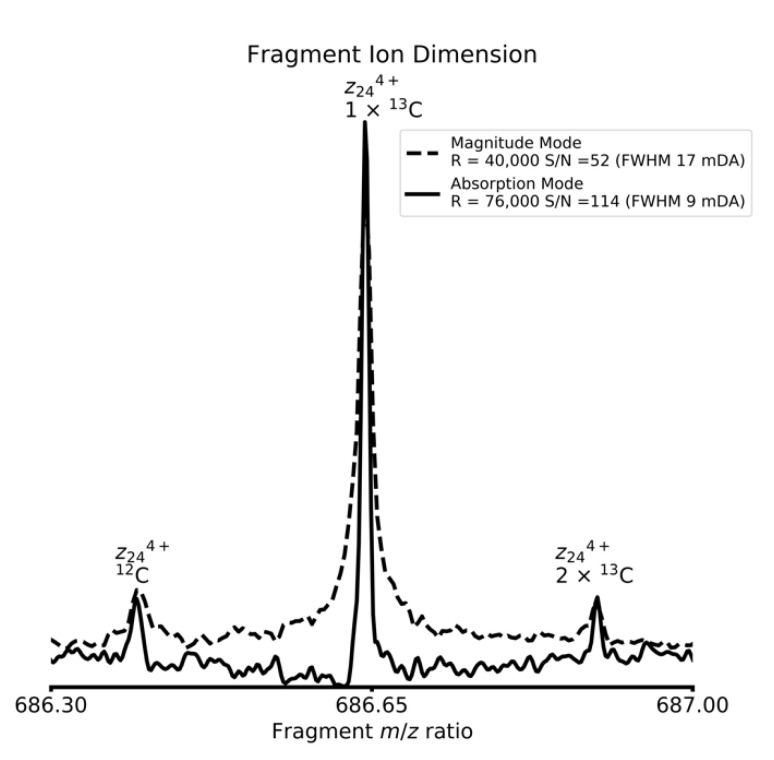
Extracted fragment ion scan from the 2D mass spectrum for the 1 × ^13^C isotope of [M + 6H]^6+^ ions of the monomethylated histone peptide (K7 1m) at *m/z* 489.453, zoom-in on fragment ion *z*_24_^4+^, comparison between absorption mode and magnitude mode.

Figure 2 focuses on the extracted fragment ion scan of the 1 × ^13^C isotope of [M+6H]^6+^ ions of the monomethylated histone peptide (K7 1m). The fragment *z*_24_^4+^ contains 24 out of the 26 residues of the peptide. Consequently, if the precursor ion contains exactly one ^13^C, then the probability of *z*_24_^4+^ retaining the ^13^C is very high [11,25,49,50,51]. In Figure 2, the relative intensity of the 1 × ^13^C isotope of *z*_24_^4+^ is much higher than the relative intensity of the ^12^C isotope. We can also see a peak for the 2 × ^13^C isotope of *z*_24_^4+^, which is caused by scintillation noise from the fragment of the 2 × ^13^C isotope of the precursor ion. The isotopic distribution shown in Figure 2 is a consequence of the precursor ion signals being isotopically resolved in the vertical dimension. In the horizontal dimension, the phase-corrected absorption mode has doubled the resolving power and signal-to-noise ratio of the magnitude mode, as expected [26,27].

**Figure 3:**
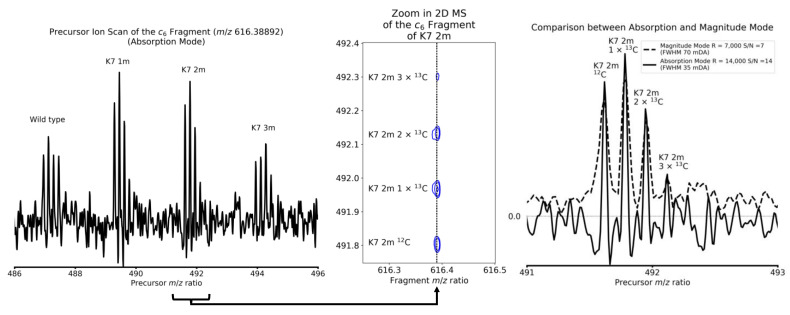
Precursor ion scan extracted from the absorption mode 2D mass spectrum for the ^12^C isotope of the *c*_6_ fragment of the histone peptides (*m/z* 616.38892), zoom-in on the *c*_6_ fragment ion isotopic distribution in the 2D mass spectrum for the dimethylated histone peptide (K7 2m), comparison between the absorption mode and magnitude mode of the precursor ion scan of the *c*_6_ fragment.

Figure 3 shows the extracted precursor ion scan of the ^12^C isotope of the *c*_6_ fragment of the histone peptides. Since the modifications are all on the 7th residue from the N-terminus, all histone peptides in the sample have identical *c*_6_ fragments. This precursor ion scan is therefore an appropriate column of the data set to test the linear phase correction function. Equations (4) and (5) predict that the phase correction function has a slope of *f*_N_*T*_1_, in which *f*_N_ is the Nyquist frequency in the vertical dimension (i.e., 10 kHz in the present experiment) and *T*_1_ is the duration of the first pulse (i.e., 739 μs). Therefore, the theoretical first order phase correction should be of 7.39 turns over the whole spectrum. The resulting spectrum is well-phased, but we observed that 7.8 turns give slightly better results (see Appendix A). Figure 3 (left) shows that the linear phase correction function yields a well-corrected spectrum in the vertical dimension. A comparison with the extracted precursor ion scan from the magnitude mode spectrum (Figure 3 right) shows that the absorption mode spectrum has doubled the resolving power and signal-to-noise ratio compared to the magnitude mode spectrum, as is predicted [26,27]. However, as this experiment was acquired in narrowband mode vertically, the frequency bandwidth is about twenty times narrower than in broadband 2D mass spectra, which may lead to a different phase correction [25].

The zoom-in on the isotopic distribution of the *c*_6_ fragment of the dimethylated histone peptide (Figure 3 centre) shows that the peaks are baseline- resolved. The full-width at half-maximum (FWHM) of the peaks is approximately 35 mDa (*R* = 14,000 at *m/z* 490), which is sufficient to isotopically resolve the fragments of biomolecules up to 14 kDa.

**Figure 4:**
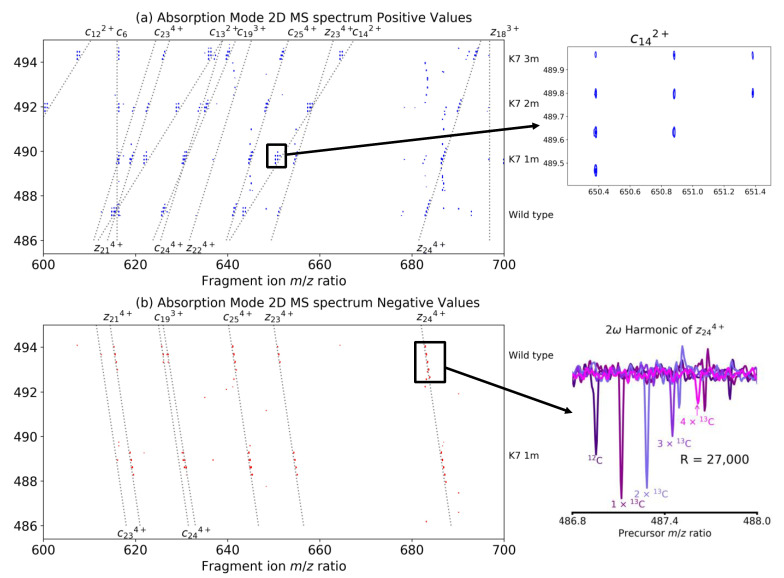
Phase-corrected absorption mode 2D mass spectrum of histone peptides, zoomed in between *m/z* 600-700 horizontally and *m/z* 485-495 vertically, (**a**) positive values plotted (insert: zoom-in on the isotopic distribution of the *c*_14_^2+^ fragment of the methylated histone peptide) and (**b**) negative values plotted (insert: vertical precursor ion scans of the isotopic distribution of *z*_24_^4+^ of the unmodified histone peptide, with frequency-to-mass conversion for the 2ω harmonic).

Figure 4a shows a zoom-in on the 2D mass spectrum in phase-corrected absorption mode with only the positive values in the 2D mass spectrum. A similar 2D mass spectrum of the same sample was analyzed in detail in a previous article [25]. We notice that all isotopic distributions are isotopically resolved in both dimensions. An insert with a zoom-in on the isotopic distribution of the *c*_14_^2+^ fragment of the monomethylated histone peptide shows that clearly. Figure 4a shows mostly fragment ion peaks with very few artefacts. Figure 4b shows the negative values in the same region, which correspond to the 2ω harmonic of the ion signal in the vertical dimension [14]. The modulation frequency of the unmodified and the monomethylated peptides is between 146.34 and 147.34 kHz.

Since the frequency range is 10 kHz, this leads to 14 foldovers for these signals. Because of the amplitude modulation of the signal in 2D MS and the real Fourier transform used during processing along the vertical axis, every time a signal is folded over in the 2D mass spectrum, the phase is shifted by 180° (or π radians). In this experiment, the signal has been folded over 14 times, and the phase is shifted by a multiple of 360°. The frequencies for the 2ω harmonic are between 292.68 and 294.68 kHz, so the signal has been folded over 29 times. Because the signal has been folded over an odd number of times, the 2ω harmonic of each dissociation line is a decreasing hyperbola, but over an *m/z* 3 range, it may appear linear [11]. The phase of the 2ω harmonic has been shifted by 29 times 180°, and the peaks corresponding to the 2ω harmonic have negative intensities, which simplifies data visualization and interpretation of the 2D mass spectrum.

The insert in Figure 4b shows the 2ω harmonic peaks for the isotopes for the *z*_24_^4+^ fragment of the unmodified histone peptide with frequency-to-mass conversion that has been adapted to the 2ω harmonics. An additional phase correction of −33° was applied to the extracted precursor ion scans to correctly phase the 2ω harmonic peaks. As has been discussed in a previous article, the harmonic signals in a 2D mass spectrum can be used to increase the resolving power, just like they can in one-dimensional mass spectra [25,52,53,54,55]. Here, we almost double the resolving power from *R* = 14,000 to *R* = 27,000.

**Figure 5:**
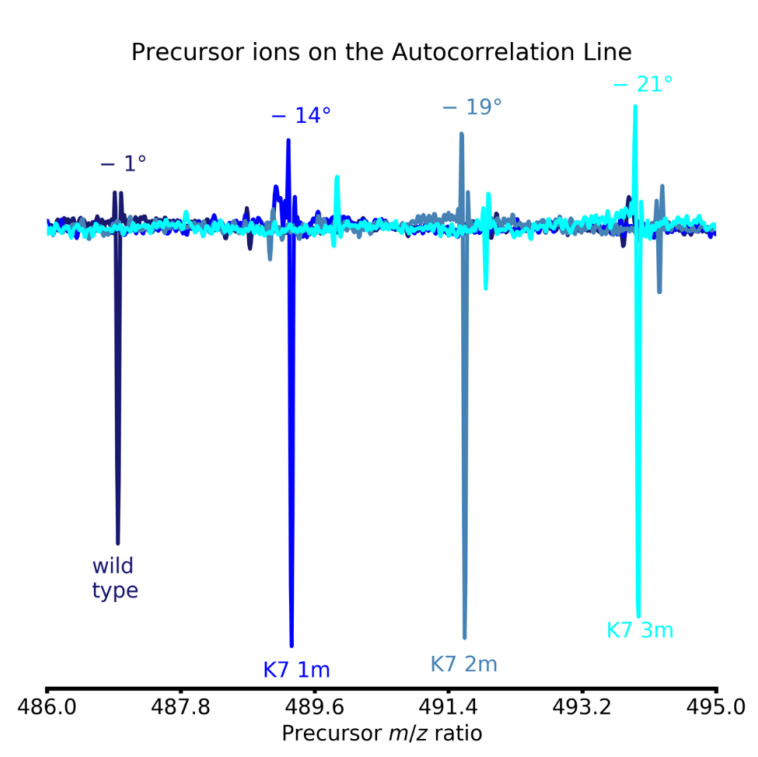
Extracted precursor ion scans for the ^12^C isotopes of the four histone peptides from the phase-corrected absorption mode 2D mass spectrum.

Figure 5 shows the precursor ion scans for the ^12^C isotopes of all four histone peptides on the autocorrelation line of the 2D mass spectrum. A comparison between equations (2) and (4) shows that, in the horizontal fragment ion dimension, the phase of precursor ion signals behaves differently from the phase of fragment ion signals, and that the phase correction function for precursor ion signals depends on *t*_1_. Furthermore, as shown in equations (2) and (3), in the vertical precursor ion dimension, the phase of precursor ion signals is shifted by −180° compared to the phase of fragment ion signals [38]. Figure 5 confirms that, with phase correction functions that aim to correct the phases of fragment ion signals, precursor ion peaks on the autocorrelation line have negative intensities, and that their phase shifts with their cyclotron frequency (and their *m/z* ratio).

## 5. Conclusions

In this study, we have for the first time obtained a phase-corrected absorption mode two-dimensional mass spectrum by performing a quadratic phase correction in the horizontal fragment ion dimension and a linear phase correction in the precursor ion dimension. The resulting 2D mass spectrum shows the expected performance improvements in terms of resolving power and signal-to-noise ratio [26,27,38]. An added benefit of phase correction is the clear differentiation between “ground state” ion signals and folded-over harmonics, which, in our case, have negative intensities. This result can be expanded to broadband 2D MS, for harmonics that have been folded over. Careful experimental design can lead to good use of this effect.

Phase-corrected absorption mode narrowband 2D MS is shown here to enable isotopically resolved tandem mass spectrometry for biomolecules up to 14 kDa on a 7 T FT-ICR mass spectrometer. This performance opens the way for the identification, location, and relative quantification, of modifications such as citrullination or de-amidation in peptides and proteins by two-dimensional mass spectrometry [25,56,57]. In future studies, phase correction for 2D MS needs to be expanded to broadband 2D mass spectra and automated for easy data processing.

## Figures and Tables

**Table 1:** Comparison between theoretically and empirically calculated coefficients for the phase correction functions in the vertical dimension (linear phase correction function) and in the horizontal dimension (quadratic phase correction function). The theoretical values are obtained from equation 28 of ref. [28], ignoring image charge effect, and ref [38]. Here, *f*_min_ is the lowest frequency in the excitation pulse and ∆*f* the frequency range in the excitation pulse, *T*_1_ is the duration of the first encoding pulse, *T*_2_ the duration of the third excitation pulse and *T*_3_ is the delay between excitation and detection, *f*_N1_ is the Nyquist frequency in the vertical precursor ion dimension and *f*_N2_ is the Nyquist frequency in the horizontal fragment ion dimension (see Scheme 1).Zero order coefficients are expressed in degrees, first and second order coefficients are expressed in turns over the whole spectrum.

Coefficient	Theory	Theoretical Value	Empirical Value
0 order (vertical)	$f_{\min}T_{1}$	88.2	59.9
1st order (vertical)	$f_{N1}T_{1}$	7.44	7.8
0 order (horizontal)	N/A	N/A	−9
1st order (horizontal)	$\frac{T_{2}f_{\min}+T_{3}\Delta f}{\Delta f}f_{N2}$	632	564
2nd order (horizontal)	$-\frac{T_{2}}{\Delta f}f_{N2}^{2}$	4607	4595

## Data Availability

Data available for download at 10.5281/zenodo.4884410.

## Sample Availability

Samples can be purchased from AnaSpec, Fremont, CA, USA.

## Appendix A

Data processing for phase correction absorption mode 2D mass spectrum

*# Import necessary modules*
import spike
from spike.File import Apex
*# Load 2D data set:*
d = Apex.Import_2D(“histonepeptide_2D_000002.d”)
*# Apodisation with a sine bell with a maximum at 15% of the transient, double zero-filling in #the horizontal dimension, Fourier transform in the horizontal dimension*
d.apod_sin(axis = 2, maxi = 0.15).chsize(d.size1,4*d.size2).rfft(axis = 2)
*# Digital demodulation by multiplying every row by*$e^{-2\pi jf_{min}t_{1}}$:
d.flipphase(0.0, 180*d.axis1.htoi(74,659.79), axis = 1)
*# Quadratic phase correction in the horizontal dimension with -9/360+564(f_2_/f_N_)+4595.7(f_2_/f_N_)^2^:**
d.phase(-9, 564.0, 4595.7, 0.0, axis = 2)
*# Discard of the imaginary part of the data in the horizontal dimension*
d.real(axis = 2)
*# Apodisation with a sine bell with a maximum at 15% of the transient, double zero-filling in the vertical dimension, Fourier transform in the vertical dimension*
d.apod_sin(axis = 1, maxi = 0.15).chsize(4*d.size1,d.size2).rfft(axis = 1)
*# Linear phase correction in the vertical dimension with 59.9/360+7.8(f_1_/f_N_):**
d.phase(59.9, 7.8, 0.0, 0.00, axis = 1)
*# Discard of the imaginary part of the data in the vertical dimension*
d.real(axis = 1)
*# Offset of the frequencies for narrowband 2D mass spectrometry*
offset = 74659.79
d.axis1.offsetfreq = 14*d.axis1.specwidth + offset
d.axis1.leftpoint = 0

* In the phase function the first variable is the zero order correction, in degrees. The second and third variable are resp. the first and second order corrections, and they are expressed in number of turns throughout the spectrum.
